# XFVM modelling of fracture aperture induced by shear and tensile opening

**DOI:** 10.1007/s10596-023-10214-5

**Published:** 2023-07-25

**Authors:** Giulia Conti, Stephan Matthäi, Patrick Jenny

**Affiliations:** 1 Institute of Fluid Dynamics, ETH Zürich, Sonneggstrasse 3, Zürich, CH-8052 Switzerland; 2 Department of Infrastructure Engineering, The University of Melbourne, Melbourne, Victoria VIC 3010 Australia

**Keywords:** Shear opening, XFVM, Fracture dilation, Fracture mechanics

## Abstract

In reservoir simulation, it is important to understand the mechanical behaviour of fractured rocks and the effect of shear and tensile displacements of fractures on their aperture. Tensile opening directly enhances the fracture aperture, whereas shear of a preexisting rough-walled fracture creates aperture changes dependent on the local stress state. Since fracture dilatation increases reservoir permeability, both processes must be included in a realistic and consistent manner into the mechanical reservoir simulation model. Here, we use the extended finite volume method (XFVM) to conduct flow and geomechanics simulations. In XFVM, fractures are embedded in a poroelastic matrix and are modelled with discontinuous basis functions. On each fracture segment the tractions and compressive forces are calculated, and one extra degree of freedom is added for both the shear and tensile displacement. In this particular XFVM implementation we assume that linear elasticity and steady state fluid pressure adequately constrain the effective stress. In this paper, shear dilation is not calculated a posteriori, but it enters the equations such that aperture changes directly affect the stress state. This is accomplished by adding shear dilation to the displacement gradients and therefore ascertains a consistent representation in the stress-strain relations and force balances. We illustrate and discuss the influence of this extra term in two simple test cases and in a realistic layer-restricted two-dimensional fracture network subjected to plausible in situ stress and pore pressure conditions.

### 1 Introduction

The aperture of fractures is an important parameter in reservoir engineering, e.g. enhanced geothermal systems (EGS) and fracking, because it influences the permeability and flow fields. In EGS, knowledge of the fracture aperture is crucial to correctly predict production [1]. Moreover, permeability enhancements through hydraulic stimulation in EGS lead to seismic events. This is reported from intermediate scale hydro shearing experiments (e.g. ISC experiment at the Grimsel test site [2]) and also from the damaging seismicity case of Basel, Switzerland [3]. Furthermore, the aperture of pre-existing fractures is not uniform in the subsurface. Therefore, preferential flow paths can emerge due to variance in the permeability field [1]. It has been shown experimentally and computationally by [4, 5] that these channelling flow paths on a single fracture plane are generated through shear processes and dilation. In EGS, these channelling flows created through shear dilation can strongly affect the heat extraction performance [6]. These examples show the importance of investigating the geomechanics of EGS with numerical models and of describing the aperture as accurately as possible for safety and production predictions.

In hydraulic fracturing, permeability enhancements are mainly driven by fracture propagation through tensile failure. Contrarily, in EGS it is assumed that the enhancement mechanism is a mixed mechanics of tensile- and shear opening of pre-existing fractures and even fracture propagation [7, 8]. In this paper, we focus on shear- and tensile opening effects.

✉ Giulia Conti
gconti@ethz.ch

Stephan Matthäi
stephan.matthai@unimelb.edu.au

Patrick Jenny
jenny@ifd.mavt.ethz.ch

1 Institute of Fluid Dynamics, ETH Zürich, Sonneggstrasse 3, Zürich CH-8052, Switzerland

2 Department of Infrastructure Engineering, The University of Melbourne, Melbourne, Victoria VIC 3010, Australia

Shear opening is created through shear displacement of pre-existing fractures and the mismatch of wall asperities. The latter leads to irreversible fracture openings and maximum closure dependent on roughness and wall strength [9]. In experiments, shear opening is observed to be non linear and to have a higher opening rate at the start of shearing [10–12]. Constitutive models describe shear opening (or shear dilation) as a function of shear displacement and dilation angle while their main difference is the definition of the latter. Willis-Richards et al. [13] and Rahman et al. [14] assume the dilation angle to be a function of the effective stress and a constant surface roughness. With decreasing effective stress (i.e. higher shear displacement), the dilation angle increases, leading to a bigger shear dilation. McClure and Horne [15] expanded this approach by two separate dilation angles to take into consideration that with increasing slip the additional shear dilation per shear displacement is smaller. Barton et al. [9] describe the dilation angle with the joint roughness coefficient and joint compressive strength. They include the non linear opening effect with a continuous reduction in dilation angle after a peak value to model the abrading asperities. In contrast to these semi-empirical models, Kotousov et al. [16] developed a theoretical model to describe roughness induced shear opening.

In numerical geomechanics simulations, numerous authors take into account changes in the aperture, but only to adapt the permeability. For example, in the extend finite element method (XFEM), Ren et al. [17] adapt the permeability by calculating the aperture from the displacement jump. In the assumed enhanced strain (AES) model, Liu [18] accounts for fracture opening by using the cohesive fracture model. Cusini et al. [19] consider normal opening and adjust the hydraulic aperture by a constant correction term in case of contact to account for rough surfaces (embedded FEM with AES). Many authors specifically include shear dilation and normal closure (compliance) in the hydraulic aperture. For example, Azizmhammadi and Matthäi [20] (FEM) and Ucar et al. [21] (discrete fracture model, DFM) use the Barton and Bandis model [9] for shear dilation, while also accounting for normal closure in their permeability modelling. Most of these authors, however, apply constant shear dilation angles, as for example Ghaderi et al. [22], who combine XFEM with the discrete element model (DEM) and calculate permeability changes through normal compliance and shear dilation. In addition to these two effects, Kohl and Mégel [23] (flow solver HEX-S), Zhang et al. [6] (discrete fracture Network, DFN), and Norbeck et al. [24] (embedded fracture model, EFM) take into account tensile opening in their flow solver to adapt the hydraulic permeability. Instead of a constant dilation angle, Deb and Jenny [25, 26] (XFVM) use an asymptotic curve model to describe shear dilation and also include tensile opening. However, in all of the listed models the effect of shear dilation on the mechanics, i.e., on the stress field, is neglected.

Rivas et al. [27] include irreversible linear shear dilation in the contact model of XFEM and simulate slip and dilation of two perpendicular fracture sets. Steffanson et al. [28] couple shear dilation with the stress in context of a mixed-dimensional DFM model by adapting the gap functions by including shear dilation which enters their deformation constraints. They report a possible significant influence of shear dilation on the mechanics, especially a reduced shear displacement originating from increased normal tractions. To the best of our knowledge, this effect has not yet been introduced in embedded fracture models, nor it has been quantified, and the implications for a fracture pattern have not yet been investigated.

In this paper, we use the extended finite volume method (XFVM) to simulate the local deformations of a porous fractured rock. XFVM was introduced by Deb and Jenny [25, 29] for shear failure and poroelastic matrices. Later the model was extended for tensile opening of fractures [26], for fracture propagation [30], and with smoothening of the shear stresses to reduce oscillations [31]. In XFVM, the fractures are lower dimensional manifolds embedded in a matrix domain. The traction and tension forces are calculated on each fracture segment to check if shear displacement or tensile opening occurs. This leads to a piecewise constant displacement along the embedded fractures.

As mentioned, a change in aperture affects the displacement and therefore also the stress field. We extended the XFVM formulation by including shear opening directly in the description of the domain displacement and hence also in the stress strain relation and force balance which is solved. This is achieved by adding a linear constitutive relation between shear slip and shear opening (simplification of [13]), and without adding additional degrees of freedom or basis functions.

The paper is structured as follows: In Section 2, we describe the governing geo-mechanical equations, the XFVM discretization and the new addition of shear opening into the model. In Section 3, the influence of shear opening on the geo-mechanics is investigate within simple test cases of a single fracture and parallel fractures, and a more complex one consisting of around 200 joints [32]. Finally, the results are discussed and summarised in Section 4.

### 2 Theory

In the following, we present the governing equations for the geomechanics of an elastic fractured rock (Section 2.1), the

XFVM discretization including shear slip and tensile opening (Section 2.2), and the addition of shear opening in the XFVM framework (Section 2.3).

#### 2.1 Equations for Geomechanics

In the matrix domain, the governing equation is the force balance

$$\nabla \cdot \boldsymbol{\sigma} + \mathbf{f} = 0, \tag{1}$$

where $\boldsymbol{\sigma}$ is the stress tensor and $\mathbf{f}$ the volume forces. The latter are assumed to be zero in the following. Furthermore, we assume linear elasticity of the rock, that is,

$$\boldsymbol{\sigma} = \lambda(\nabla \cdot \mathbf{u})\mathbf{I} + G(\nabla \mathbf{u} + \nabla \mathbf{u}^T), \tag{2}$$

where $\mathbf{u}$ is the displacement vector, $\lambda$ and $G$ the first and second Lamé constant, and $\mathbf{I}$ the identity matrix. The normal compressive stress and shear traction on each fracture segment are

$$\sigma_c = -\hat{\mathbf{n}} \cdot (\boldsymbol{\sigma} \cdot \hat{\mathbf{n}}) \tag{3}$$

and

$$\tau = \hat{\mathbf{t}} \cdot (\mathbf{I} - \hat{\mathbf{n}} \cdot \hat{\mathbf{n}}^T) \cdot (\boldsymbol{\sigma} \cdot \hat{\mathbf{n}}), \tag{4}$$

respectively, where $\hat{\mathbf{n}}$ and $\hat{\mathbf{t}}$ represent the unit normal and tangent vector. Shear displacement occurs if $\tau$ exceeds the shear strength $\tau_{\max}$. In [29] it is described that relaxation of $\tau_{\max}$ is needed for grid convergence in XFVM. This shear relaxation was proposed by Prakash and Clifton [33] and is denoted by

$$\frac{\mathrm{d}\tau_{\max}}{\mathrm{d}t} = -\frac{1}{t^f_{PC}}(\tau_{\max} - \mu_s(\sigma_c - p^f)). \tag{5}$$

The parameter $t^f_{PC}$ is the shear relaxation timescale, $\mu_s$ is the friction coefficient and $p^f$ is the fluid pressure. Note that for $t^f_{PC} \rightarrow \mathrm{d}t$ the Coulomb criterion is recovered. If shear failure occurs, i.e., if $\tau > \tau_{\max}$, then the criterion $\tau = \tau_{\max}$ is enforced. Similarly, if tensile opening occurs, i.e., if $p^f > \sigma_c$, then the two criteria $\sigma_c = p^f$ and $\tau = 0$ are enforced.

In this study we assume the fluid pressure to be uniform over the fractured rock, i.e., the pressure in the porous rock and the fractures is equal. Hence, we do not discretize the flow field, but simulate stress states and displacement fields for different uniform fluid pressures. This is comparable to performing mechanical simulation of a fracture pattern in different depths below the earth's surface subjected to different pressure conditions.

#### 2.2 XFVM-discretization

The extended finite volume method introduced by Deb and Jenny [25, 29] is employed to solve the stress equilibrium over the fractured matrix domain. We integrate the force balance over the domain $\Omega$ leading to

$$\int_{\partial\Omega} \boldsymbol{\sigma} \cdot \hat{\mathbf{n}}\, \mathrm{d}A = \int_{\partial\Omega} (\lambda(\nabla \cdot \mathbf{u})\mathbf{I} + G(\nabla \mathbf{u} + \nabla \mathbf{u}^T)) \cdot \hat{\mathbf{n}}\, \mathrm{d}A = 0 \tag{6}$$

The displacement vector $\mathbf{u}(\mathbf{x})$ at point $\mathbf{x}$ in the matrix domain can be approximated by the ansatz

$$\mathbf{u}(\mathbf{x}) \approx \sum_{I=1}^{N_e} \mathbf{u}_I N_I(\mathbf{x}) + \sum_{J=1}^{N_s} \hat{\mathbf{t}}_J s^J_\tau N^J(\mathbf{x}) + \sum_{J=1}^{N_s} \hat{\mathbf{n}}_J s^J_n N^J(\mathbf{x}). \tag{7}$$

In Equation (7), $\mathbf{u}_I$ denotes the displacement at the grid point $\mathbf{x}_I$ and $N_I(\mathbf{x})$ is its corresponding basis function which is composed of four bilinear interpolation functions; see Fig. 1 showing the boundary (dashed line) of a finite volume $\Omega_I$ of which the forces are calculated.

The fractures are represented by sets of 1D elements in a 2D matrix domain, and the displacement jump across a fracture manifold is accounted for by the second and third terms on the right hand side of Equation (7). For each fracture segment, two degrees of freedom (dof) are added; one for the shear displacement $s^J_\tau$ and one for the tensile opening $s^J_n$ (acting in tangent and normal direction, respectively). Here, each fracture segment is bounded by a grid cell, see Fig. 1.

The special discontinuous basis function $N^J(\mathbf{x})$ is defined as

$$N^J(\mathbf{x}) = \sum_{p=1}^{N_e} N^J_p(\mathbf{x}) H^J_p(f^J(\mathbf{x})) \tag{8}$$

with

$$H^J_p(f^J(\mathbf{x})) = H(f^J(\mathbf{x})) - H(f^J(\mathbf{x}_p)), \tag{9}$$

where $H(.)$ is the Heaviside function and $f^J(.)$ is the signed distance from fracture segment $J$ to point $\mathbf{x}$ or grid node $\mathbf{x}_p$. For the special basis function, $N^J(\mathbf{x}) \neq 0$ holds true only if grid node $\mathbf{x}_p$ is a neighbour of fracture segment $J$.

The basis functions are defined such that the displacement gradient is continuous across the fracture. Hence, no additional modelling criteria are needed to calculate the shear ($\tau$) and normal ($\sigma_c$) stresses acting on the fracture segments.

**Fig. 1** Continuous basis function $N_I$ and finite volume $\Omega_I$ on the left and special basis function $N^J$ and finite volume $\Omega_I$ in the case of a fracture manifold on the right. The displacement degrees of freedom for the grid and fracture segments are marked by black points

When shear slip or tensile opening occurs, the additional constraints are imposed in an integral sense on each fracture segment of length $L$ as

$$\int_0^L \tau \, \mathrm{d}l = L\tau_{\max} \tag{10}$$

for shear slip and

$$\int_0^L \tau \, \mathrm{d}l = 0 \quad \text{and} \quad \int_0^L \sigma_c \, \mathrm{d}l = \int_0^L p^f \, \mathrm{d}l \tag{11}$$

for tensile opening, respectively. Equations (6), (10), and (11) are solved to obtain the dofs $\mathbf{u}_I$, $s_\tau^J$, and $s_n^J$. If multiple fractures are present in a grid cell, a merging technique is applied where segments in a cell are merged and the weakest link is employed (Conti et al. (2022) under review).

### 2.3 XFVM-discretization with shear opening

Shear opening is the dilation of a fracture created through shear displacement and the surface roughness of the fracture; see Fig. 2.

Since we assume linear elastic behaviour, we apply a model describing shear opening as proposed by Willis-Richards [13]. It reads

$$b(s_\tau^J) = |s_\tau^J| \tan(\phi_{\mathrm{dil}}^{\mathrm{eff}}), \tag{12}$$

where $\phi_{\mathrm{dil}}^{\mathrm{eff}}$ is the effective dilation angle. The latter is a function of the normal effective stress $\sigma_c$ and the dilation angle $\phi_{\mathrm{dil}}$. The dilation angle is a laboratory measured rock property describing roughness. Equation (12) has been widely used in the literature and values employed for $\phi_{\mathrm{dil}}$ vary between $1° - 5°$ [6, 14, 23, 24, 28]. Willis-Richards et al. [13] refer to 1.5° as a "smooth" and to 5° as a "fairly smooth" surface.

Other models for shear opening and the dilation angle are for example described by McClure and Horne [15], where the Willis-Richards approach is extended, or by Barton and Bandis [9], where the dilation angle is a function of the effective stress and mobilized joint roughness coefficient. In this paper, we assume $\phi_{\mathrm{dil}}^{\mathrm{eff}} = \phi_{\mathrm{dil}}$ for simplicity; the dilation angle is assumed to be constant and is set to $\phi_{\mathrm{dil}} = 5.7°$ to simulate a rougher surface. Consequently, shear opening $b(s_\tau^J)$ is linearly dependent on the shear slip $s_\tau^J$.

Shear opening is a displacement in the normal direction to the fracture manifold. This opening also induces a volume change, which we include in the stress strain relation by adapting the displacement approximation to

$$\mathbf{u}(\mathbf{x}) \approx \sum_{I=1}^{N_e} \mathbf{u}_I N_I(\mathbf{x}) + \sum_{J=1}^{N_s} \hat{\mathbf{t}}_J s_\tau^J N^J(\mathbf{x}) + \sum_{J=1}^{N_s} \hat{\mathbf{n}}_J (s_n^J + b(s_\tau^J)) N^J(\mathbf{x}). \tag{13}$$

Opposed to approximation (7), here the shear dilation effect is included. When tensile opening starts, the fractures are not in contact any more, and no additional shear opening will develop. For this reason, as soon as tensile opening appears, i.e., when $p^f > \sigma_c$, shear opening is fixed to the current value $b_{\tau,\max}^J = b(s_\tau^J)$. For the aperture $b^J$ of segment $J$ this

**Fig. 2** Schematic of shear opening $b(s_\tau)$ due to asperities and shear displacement $s_\tau$. Adapted from [14]

can be summarized as

$$b^J = \begin{cases} a_0^J + b(s_\tau^J), & \text{if } p^f < \sigma_c \\ a_0^J + b_{\tau,\max}^J + s_n^J, & \text{otherwise,} \end{cases} \tag{14}$$

where $a_0^J$ is the initial aperture of the fracture. This opening can represent previous deformations of the fracture and in general is different for every segment $J$. In this paper we do not include normal compliance (elastic fracture behaviour, where $a_0$ depends on the effective normal stress) in Equation (14). Furthermore, shear dilation is an irreversible process, and therefore $\Delta b(s_\tau^J) > 0$ has to be enforced.

The shear slip has a signed direction in our model, but shear opening is not dependent on the slip direction itself, which is why we take its absolute value in Equation (12). Initially when the fracture segment slips, its slip direction is not known. For this reason, we neglect shear opening during the first iteration step and if $s_\tau = 0$, i.e., $b(s_\tau)$ is set to zero. After the first iteration step, the shear slip has established its direction which is then needed to correct the sign in Equation (12). For later iterations $k > 0$, the sign correction depends on the slip direction at the previous iteration $k - 1$.

In the linear system solved, the additional shear opening term increases the non diagonal coefficients in the linear equations for $\mathbf{u}_I$, Equation (6), and increases the diagonal coefficients in the linear equations for the dofs $s_\tau$, Equation (10). In a test case with a single fracture this leads to slightly better grid convergence (Appendix A). If the dilation angle is not constant, but a function of compressive stress, then these coefficients need to be adapted after each time step.

In this paper, we do not distinguish between hydraulic and void aperture. One could, however, introduce two different apertures and dilation angles. In that case, the void aperture affects the stresses. On the other hand, the hydraulic aperture is important for the permeability. We assume a constant dilation angle, but the model can easily be extended for stress dependent dilation angles.

**Fig. 3** Setup: Inclined fracture in 2 km × 2 km domain with 100 × 100 cells on the left and an enlargement of the upper tip region on the right. The fracture's start and end coordinates are $x_s = 0.65$ m, $y_s = 0.5337$ m, $x_e = 1.36$ m, $x_e = 1.59$ m

## 3 Results

### 3.1 Single Fracture

The effect of shear opening is investigated using a simple 2D plane strain testcase, where a 1.272 km long inclined (33.9° to $\sigma_y$) fracture is embedded in a 2 km × 2 km domain with a uniform grid spacing of 20 m; see Fig. 3. We apply normal compressive stress boundary conditions of $\sigma_x = 12$ MPa on the eastern and western boundaries and $\sigma_y = 18$ MPa on the southern and northern boundaries. The shear stress boundary conditions are set to 0 MPa on all four faces. The initial pressure $p^f_{init}$ in the domain (fracture and porous rock matrix) is 6 MPa and is augmented at a rate of 0.01 MPa/s. This causes the fracture to slip and open. We conducted two simulations with the same setup, but two different dilation angles: $\phi_{\text{dil}} = 0°$ and $\phi_{\text{dil}} = 5.71°$. Other parameters employed in the simulations are summarized in Table 1. The Lamé constants describe a Permian Sandstone from Annan (PS) (Table 2).

At a pressure of 9 MPa the fracture slips and therefore also dilates for the case with $\phi_{\text{dil}} = 5.71°$; see Fig. 4. We notice that there is a difference in shear displacement between the two cases; if shear opening is accounted for, the shear displacement is slightly (-3.6%) smaller. This effect can be explained using the compressive stress and shear stress acting on the fracture (Fig. 4). The compressive stress for $\phi_{\text{dil}} = 5.71°$ is higher than for $\phi_{\text{dil}} = 0°$. A higher compressive stress leads to an increased shear strength $\tau_{\max}$ (Equation 5) and thus to smaller displacements. This confirms the results by Steffanson et al. [28].

Figure 5 shows shear slip, tensile opening and aperture for $p^f = 9$, 11, 13, and 15 MPa. At $p^f = 9$ MPa, no tensile opening is present, and therefore the aperture change $\Delta b = b - a_0$ is only due to shear opening. At $p^f = 11$ MPa and 13 MPa, small tensile opening can be observed at the fracture tails. See Appendix B for the matrix displacement and mean stress at $p^f = 11$ MPa. At $p^f > 14$ MPa, tensile opening is significant along the whole fracture as the pressure exceeds

**Table 1** Parameters

| Model parameter | Value |
|---|---|
| Boundary stress $\sigma_x$ | 12 MPa |
| Boundary stress $\sigma_y$ | 18 MPa |
| Initial fluid pressure $p^f_{init}$ | 6 MPa |
| Friction coefficient $\mu_s$ | 0.46 |
| Initial fracture aperture $a_0$ | 1 mm |
| Timescale $t^f_{PC}$ | 10 s |
| Time step $dt$ | 10 s |
| Pressure increase per $dt$ | 0.01 MPa/s |

**Table 2** Lamé constants for Sandstones as proposed in [34] and [35]

| Rock type | Name | **λ** [GPa] | ***G*** [GPa] |
|---|---|---|---|
| Middle Devonian Sandstone from Dounreay | MDS | 12.60 | 13.80 |
| Permian Sandstone from Annan | PS | 6.30 | 6.90 |
| Upper Carb Sandstone from North Devon | UCS | 4.00 | 18.00 |

the normal compressive stress (Fig. 6). We see that tensile opening exceeds shear opening nearly immediately after (at $p^f = 14.3$ MPa). At $p^f = 15$ MPa the two cases $\phi_{\text{dil}} = 0°$ and $\phi_{\text{dil}} = 5.71°$ lead to the same shear slip $s_\tau$ and aperture change $\Delta b$ but different tensile openings $s_n$ (Fig. 5). This means that if shear opening is not accounted for ($\phi_{\text{dil}} = 0°$), the effect of it will be included in the tensile opening dof $s_n$ for $p^f > \sigma_c$. Note that $\tau = 0$ is enforced during tensile opening. We conclude that if $p^f < \sigma_c$ shear opening is needed to capture the aperture change through slip, whereas for $p^f > \sigma_c$ the effect is captured in $s_n$ even for $\phi_{\text{dil}} = 0°$.

We note that the not smooth slip, aperture, and stress profiles in Figs. 4 and 5 originate from inclined embedded fractures in XFVM. We refer to Behbahani et al. [31], who solve this issue by smoothening the stress at every iteration step and therefore achieve smooth shear slip profiles along the fracture.

### 3.2 Parallel fractures

The next example is a test case of two parallel fractures; see Fig. 7. The same boundary conditions and simulation parameters (Table 1) were used as in Section 3.1. The rock is a Middle Devonian Sandstone from Dounreay (Table 2). The fractures have a length of 0.93 km, are oriented at an angle of 33.9° to the normal boundary stress $\sigma_y$, have a spacing of 25.16 m and overlap over 506 m. We investigate the results at $p^f = 9$ MPa.

As with the single fracture, shear displacement is slightly lower in the parallel fractures, if the coupled shear opening is accounted for (Fig. 8). However, the relative difference varies over the fracture. In the region of overlap, the difference is around −5%, whereas it decreases to −3.5% in the region without overlap. As a comparison, if only the right fracture is present, the relative change in shear slip is around −4.3% in the middle of the fracture; see Fig. 9. The relative difference profiles are subjected to fluctuations and high values at the fracture ends because of the small shear slip values and higher stress fluctuations (similar as in Fig. 5). Nevertheless, a clear trend in relative change is observable in the middle part of the parallel fractures. This simple example of two fractures clearly shows that the shear opening influences the shear displacement behaviour of the fractures in its proximity.

### 3.3 Natural fracture pattern

The influence of shear opening is investigated within a fracture pattern. To this end we use a fracture pattern mapped and described by [32, 34]. It originates from a highly fractured Middle Devonian sandstone mapped in Dounreay, Scotland. The fractures are randomly oriented creating polygonal fractures. The boundary conditions imposed are compressive stresses $\sigma_{e,x} = 12$ MPa and $\sigma_{n,y} = 18$ MPa on the eastern and northern faces, respectively. On the western and southern boundaries we impose displacement conditions of $u_{w,x} = 0$ mm and $u_{s,y} = 0$ mm. On all four faces the shear stresses are 0 MPa. Parameters employed for the simulations are described in Table 1. The Lamé constants were proposed

**Fig. 4** Shear slip $s_\tau$, change in aperture $\Delta b$, shear traction $\tau$, and compressive stress $\sigma_c$ along fracture for $\phi_{\text{dil}} = 0°$ (solid lines) and $\phi_{\text{dil}} = 5.71°$ (dashed lines) at $p^f = 9$ MPa

**Fig. 5** Shear slip $s_\tau$, tensile opening $s_n$, aperture $b$, shear stress $\tau$, and compressive stress $\sigma_c$ along fracture at $p^f = 9$ MPa, $p^f = 11$ MPa, $p^f = 13$ MPa, and $p^f = 15$ MPa. The dashed lines indicate values for $\phi_{\text{dil}} = 5.71°$ and the continuous lines for $\phi_{\text{dil}} = 0°$

by [34] and describe a Middle Devonian sandstone (MDS) from Dounreay (Table 2). The domain size is 2.24 m × 2.28 m and the pattern is embedded in a 56 × 57 Cartesian grid. The results are shown for a fluid pressure of 11 MPa.

Figures 10 and 11 show shear displacement and aperture along the fractures for the dilation angles $\phi_{\text{dil}} = 0°$ and $\phi_{\text{dil}} = 5.71°$, as well as the relative difference. The latter is defined as

$$\% \text{ SC} = \frac{s_\tau(\phi_{\text{dil}} = 5.71) - s_\tau(\phi_{\text{dil}} = 0)}{s_\tau(\phi_{\text{dil}} = 0)} \times 100 \qquad (15)$$

with a threshold of $s_\tau \geq 0.005$ mm for the slip to exclude very high relative changes originating from very small values.

The relative aperture difference is calculated analogously. Shear slip and aperture are notably different in some regions because of the added effect of shear opening. Interestingly, the relative shear slip change is negative in some and positive in other regions, notably varying between −50% and 50%.

The aperture change is mostly positive in fractures subjected to shear slip (max of 3.4%), but also negative in some segments (min -4.9%). Negative changes are caused by the fracture pattern mechanics, since a change at one place affects the whole domain. Furthermore, we note that they do not originate from fracture closure for $\phi_{\text{dil}} = 5.71°$, but from tensile

**Fig. 6** Aperture change due to shear opening and tensile opening at center point of fracture after pressure increase for $\phi_{\text{dil}} = 5.71°$

**Fig. 7** Setup of parallel fractures embedded in a 2 km × 2 km domain with 100 × 100 grid cells

**Fig. 8** Shear slip and relative difference profiles of $\phi_{dil} = 0°$ and $\phi_{dil} = 5.71°$ along the left and right fractures of Fig. 7

opening for $\phi_{\text{dil}} = 0°$, which is not present for $\phi_{\text{dil}} = 5.71°$. The opposite is true in other segments, that is, tensile opening might be present for $\phi_{\text{dil}} = 5.71°$ and not for $\phi_{\text{dil}} = 0°$; see Fig. 12. Hence, contrarily to the case of a single fracture, including shear opening in fracture networks does not always lead to a decrease in shear slip and an increase in aperture. We conclude that the overall mechanical behaviour can differ considerably in the domain, if shear opening is taken into account.

For half the size of $\lambda$ and $G$ (sandstone PS in Table 2) the effects on shear slip and aperture are even more pronounced (Figs. 13 and 14). For the sandstone UCS (Table 2) the mechanical behaviour is different; most noticeable is the opposite sign of $\%s_\tau$ for some fracture segments compared to MDS ($\lambda$ = 12.6 GPa, $G$ = 13.8 GPa) and PS ($\lambda$ = 6.3 GPa, $G$ = 6.9 GPa). The different relative changes in $s_\tau$ and $b$ among the three rocks shows rock type dependent behaviour.

**Fig. 9** Test case with only the right fracture of Fig. 7 (one single fracture in matrix domain). Shear slip and relative difference profile along fracture length

### 4 Conclusion

In this paper, we included the effect of shear opening directly in our approximation of the displacement field and therefore in the stress and force balance solved with an embedded fracture model. Hence the volume change generated through shear displacement is now directly affecting the mechanics. This is done without adding new degrees of freedom or basis functions. A simple constitutive relation between shear slip and shear dilation was chosen, but the model can be extended for stress dependent relations. A test case of a single inclined fracture in the domain shows that shear displacement is lower, if shear opening is included. The effect is around 4% for the sandstone investigated. The reason for this is that the compressive stresses on the fracture and thus the shear strength increase.

Contrarily, if shear opening is not included, then tensile opening is slightly higher, which leads to the same total aperture and shear displacement in the fracture. However, tensile opening is activated at higher fluid pressures than shear opening, meaning that an aperture change at lower pressures is not captured if the shear opening effect is neglected. These results show that in fracture models where tensile opening is the main opening mechanism, shear opening could be neglected. However, in test cases where shear displacement can play an important role as e.g. in EGS, shear opening clearly has an

**Fig. 10** Shear displacements with $\phi_{\text{dil}} = 0°$ and $\phi_{\text{dil}} = 5.71°$ and difference between the two cases. The fracture segments without shear slip are indicated by light green lines

**Fig. 11** Aperture of fractures for $\phi_{\text{dil}} = 0°$ and $\phi_{\text{dil}} = 5.71°$ and difference between the two cases. The fracture segments without aperture change are indicated by light green lines

**Fig. 12** Tensile opening of fractures for $\phi_{\text{dil}} = 0°$ and $\phi_{\text{dil}} = 5.71°$ and difference between the two cases. The fracture segments without aperture change are indicated by light green lines

**Fig. 13** Shear slip difference $\%s_\tau = \frac{s_\tau(\phi_{\text{dil}}=5.71°) - s_\tau(\phi_{\text{dil}}=0°)}{s_\tau(\phi_{\text{dil}}=0°)}$ for three different sandstones PS, MDS, and UCS

influence on the displacement field and needs to be accounted for.

In the natural fracture pattern investigated here, the effect of shear opening is more complex; shear displacements along fractures decrease, but also increase in other parts, showing the redistribution of displacement and stress across the domain. As a consequence of accounting for shear dilation, tensile opening starts later in some segments. Moreover, long fractures subjected to slip dilate, which can have an influence on the flow paths and overall permeability. These results suggest possible future studies; e.g. investigating different fracture patterns and rock types to better understand when shear opening is important for the mechanics. Also, investigating shear opening of a 3D fractured rock would be valuable to evaluate the aperture mechanisms in enhanced geothermal systems. We expect the challenges therein to be on the side of the discretization of intersecting fractures in XFVM, however, the shear dilation could be added in the same manner as presented in this paper.

**Acknowledgements** The authors acknowledge Rajdeep Deb for providing the legacy XFVM code and thank Michael Liem, ETH Zürich and Ranit Monga, ETH Zürich for constructive discussions.

**Author Contributions** Giulia Conti: Software, Formal Analysis, Conceptualization, Methodology, Writing - original draft. Patrick Jenny: Conceptualization, Methodology, Writing - review & editing, Supervision, Funding acquisition. Stephan Matthäi: Conceptualization, Methodology, Writing - review & editing, Supervision.

**Funding** This work was supported by the Swiss National Science Foundation (SNSF) through grant SNF 200021_178922/1. The funding sources have no involvement in the study design, the preparation of the article and decisions related to its publication. Open access funding provided by Swiss Federal Institute of Technology Zurich

### Declarations

**Conflict of interest** The authors declare that they have no conflicts of interest.

**Fig. 14** Aperture difference $\%b = \frac{b(\phi_{\text{dil}}=5.71°) - b(\phi_{\text{dil}}=0°)}{b(\phi_{\text{dil}}=0°)}$ for three different sandstones PS, MDS, and UCS

**Open Access** This article is licensed under a Creative Commons Attribution 4.0 International License, which permits use, sharing, adaptation, distribution and reproduction in any medium or format, as long as you give appropriate credit to the original author(s) and the source, provide a link to the Creative Commons licence, and indicate if changes were made. The images or other third party material in this article are included in the article's Creative Commons licence, unless indicated otherwise in a credit line to the material. If material is not included in the article's Creative Commons licence and your intended use is not permitted by statutory regulation or exceeds the permitted use, you will need to obtain permission directly from the copyright holder. To view a copy of this licence, visit http://creativecommons.org/licenses/by/4.0/.

### Appendix A: Convergence

$$s^N_{\tau,max} = s^{converged}_{\tau,max} + \frac{c}{N^q}, \tag{16}$$

## Figures and Tables

**Fig. 15:**
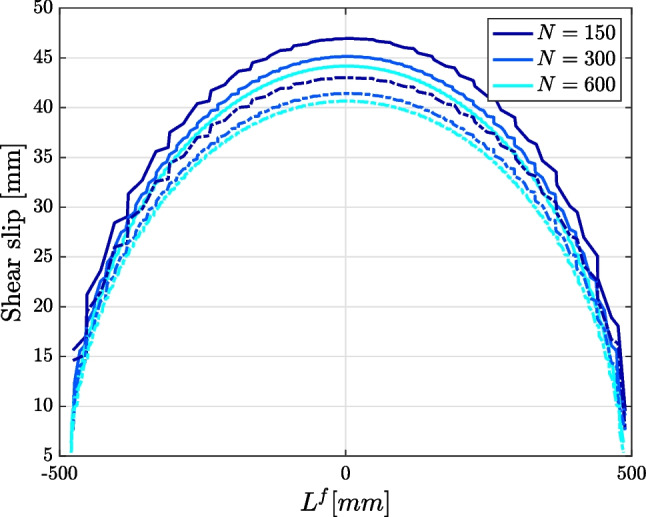
Shear slip over fracture for three different meshes $N\times N$ with $N=150$, $N=300$, and $N=600$. The continuous lines represent the case without shear dilation, i.e., with $\phi _{dil}=0^{\circ }$, and the dashed lines with shear dilation, i.e., with $\phi _{dil}=11.3^{\circ }$

**Fig. 16:**
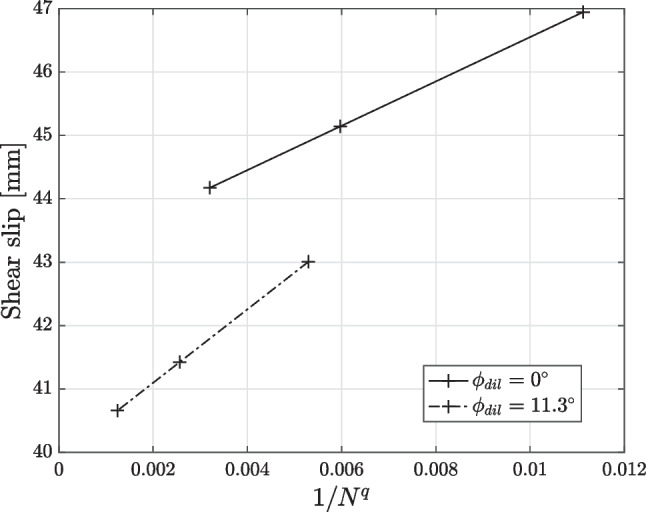
Convergence of the simulations shown in Fig. 15. For $\phi _{dil}=0^{\circ }$: $q=0.898$, $c=349.92$, and $s_\tau ^{converged}=43.1$, whereas for $\phi _{dil}=11.3^{\circ }$: $q= 1.05$, $c=579.56$, and $s_\tau ^{converged}=39.94$

**Fig. 17:**
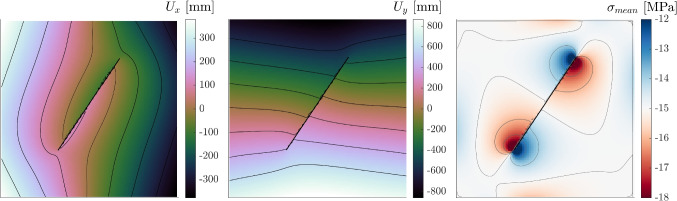
Displacement in x- and y- direction, and mean stress at $p^f={11}\,\textrm{MPa}$

## Data Availability

The datasets generated and analysed during the current study are available from the corresponding authors on reasonable request.

## Appendix A: Convergence

To investigate the influence of the additional shear dilation coupling on the convergence, we set up another test case, where the shear opening effect is very high ($\phi _{dil}=11.3^{\circ }$). The test case consists of a single fracture ($L^f={0.971}\,\textrm{km}$, $34^{\circ }$ to principle stress) embedded in a ${3}\,\textrm{km}\times {3}\,\textrm{km}$ matrix. The boundary conditions are the same as described in Section 3.1. The rock parameters are $\lambda ={12}\textrm{GPa}$, $G={14.7}\textrm{GPa}$. Figure 15 shows the shear slip profiles for meshes of $N\times N$ with $N\in \{150,300,600\}$ at $p^f={9.5}\,\textrm{MPa}$. The relative difference in shear displacement due to shear dilation in the middle of the fracture is $-8.38\%$, $-8.24\%$, and $-7.9\%$, for $N=150$, $N=300$, and $N=600$, respectively. So the refinement slightly decreases the effect of shear dilation on the shear slip.

Figure 16 shows the grid convergence for the case with and without shear opening. We are in the asymptotic limit, if the three grid spacings fulfill

16 $$\begin{aligned} s_{\tau ,max}^N=s_{\tau ,max}^{converged}+\frac{c}{N^q}, \end{aligned}$$

where *c* is the slope, *q* the convergence rate, $s_\tau ^{converged}$ the converged solution, and $s_{\tau ,max}$ the shear slips in the middle of the fracture for the three grid resolutions. Using Richardson extrapolation we get $q_{\phi =0^{\circ }}=0.898$ and $q_{\phi =11.3^{\circ }}=1.05$. So for this specific test cases, convergence is slightly better, if the coupling due to shear opening is included.

## Appendix B: Displacement and stress fields

In Fig. 17, the displacement of the matrix nodes in x- and y- directions are shown for a pressure of $p^f={11}\,\textrm{MPa}$ in the single fracture test case of Section 3.1. Only the case with shear opening is plotted, since the difference in stress and displacement for the cases with and without shear opening is too small to be seen.
